# A Personalized eHealth Intervention for Lifestyle Changes in Patients With Cardiovascular Disease: Randomized Controlled Trial

**DOI:** 10.2196/14570

**Published:** 2020-05-22

**Authors:** Eva Rosalinde Broers, Willem Johan Kop, Johan Denollet, Jos Widdershoven, Mart Wetzels, Idowu Ayoola, Jordi Piera-Jimenez, Mirela Habibovic

**Affiliations:** 1 Department of Cardiology Elisabeth-Tweesteden Hospital Tilburg Netherlands; 2 Department of Medical and Clinical Psychology Tilburg University Tilburg Netherlands; 3 University of Technology Eindhoven Eindhoven Netherlands; 4 Badalona Serveis Assistencials Badalona Spain

**Keywords:** cardiovascular diseases, lifestyle, habits, eHealth, mHealth

## Abstract

**Background:**

Behavior change methods involving new ambulatory technologies may improve lifestyle and cardiovascular disease outcomes.

**Objective:**

This study aimed to provide proof-of-concept analyses of an intervention aiming to increase (1) behavioral flexibility, (2) lifestyle change, and (3) quality of life. The feasibility and patient acceptance of the intervention were also evaluated.

**Methods:**

Patients with cardiovascular disease (N=149; mean age 63.57, SD 8.30 years; 50/149, 33.5% women) were recruited in the *Do Cardiac Health Advanced New Generation Ecosystem (Do CHANGE)* trial and randomized to the *Do CHANGE* intervention or *care as usual* (CAU). The intervention involved a 3-month behavioral program in combination with ecological momentary assessment and intervention technologies.

**Results:**

The intervention was perceived to be feasible and useful. A significant increase in lifestyle scores over time was found for both groups (*F*_2,146.6_=9.99; *P*<.001), which was similar for CAU and the intervention group (*F*_1,149.9_=0.09; *P*=.77). Quality of life improved more in the intervention group (mean 1.11, SD 0.11) than CAU (mean −1.47, SD 0.11) immediately following the intervention (3 months), but this benefit was not sustained at the 6-month follow-up (interaction: *P*=.02). No significant treatment effects were observed for behavioral flexibility (*F*_1,149.0_=0.48; *P*=.07).

**Conclusions:**

The Do CHANGE 1 intervention was perceived as useful and easy to use. However, no long-term treatment effects were found on the outcome measures. More research is warranted to examine which components of behavioral interventions are effective in producing long-term behavior change.

**Trial Registration:**

ClinicalTrials.gov NCT02946281; https://www.clinicaltrials.gov/ct2/show/NCT02946281

## Introduction

The elimination of modifiable behavioral risk factors for cardiovascular disease (eg, smoking and physical inactivity) in the general population could prevent 80% of adverse clinical outcomes [1]. In patients diagnosed with cardiovascular disease, a modest reduction in risk behaviors can decrease the mortality rates by approximately 50% [2]. However, recommended targets (eg, lifestyle and medication adherence) for secondary prevention are rarely reached [3]. To achieve sustained health behavior change, active interventions that go beyond patient education are needed [4].

Sustainable behavior changes can be enhanced by implementing a personalized patient-tailored approach [5,6]. New ambulatory technologies can now be used to provide personalized support in a low threshold, nonobtrusive, and ecologically valid manner. These devices can be used to provide feedback about ambulatory health behaviors (eg, physical activity levels), but they are not sufficient to produce long-term behavior change [7,8]. In the setting of cardiac rehabilitation, telemonitoring guidance for patients’ physical activity levels was found to be feasible in the FIT@Home study, and this intervention resulted in higher patient satisfaction and trends toward lower health care costs, but not in better improvements in fitness or physical activity levels relative to standard center-based rehabilitation [8]. The impact of this intervention could potentially have been further improved if a more patient-tailored approach were added. Another study found initial support that an app using persuasive design techniques can improve biological and psychological factors in patients after cardiac rehabilitation [9]. It is therefore plausible that ambulatory assessments are likely to have better therapeutic effects when combined with prompts that promote health-related behaviors (ie, ecological momentary interventions). These new methodologies also require a deeper knowledge about patients’ needs and preferences [10].

This trial (*Do Cardiac Health Advanced New Generation Ecosystem*, Do CHANGE) was specifically designed to examine this multidisciplinary approach to behavior change [11]. What is unique to this trial is that patients received the behavior change program, *Do Something Different* (DSD), which has been previously developed to change unhealthy habits through the increase of behavioral flexibility [11]. Behavioral flexibility is associated with a broad range of the behavioral repertoire, making people more open to experience and the adoption of new behaviors [12]. DSD has been evaluated in other patient samples and has shown promising results by producing health behavior change [13]. For this study, the program was adapted to meet the needs of patients with cardiovascular disease (coronary artery disease, CAD; heart failure, HF; and hypertension, HT). Hence, the aim of this study was to provide proof of concept for the behavioral intervention aiming to address (1) behavioral flexibility, (2) lifestyle change, and (3) quality of life. The feasibility and patient acceptance of the intervention were also evaluated.

## Methods

### Design

The Do CHANGE trial is an international (the Netherlands and Spain), multicenter, randomized controlled trial, designed to enhance lifestyle changes in patients with cardiac disease (NCT02946281). The trial findings described in this paper are the first (proof of concept and feasibility) phase of the Do CHANGE project (phase 1) and will serve as input for further development of a second phase of this randomized controlled trial (Do CHANGE, phase 2; NCT03178305). A detailed description of both phases of the Do CHANGE trial has been published previously [11]. As this trial was developed to provide information about proof of concept and feasibility, an a priori sample size calculation was not performed. For this phase, we aimed to include 150 patients across 2 countries, which is considered sufficient to give information about proof of concept and feasibility of the intervention.

### Study Sample

Patients diagnosed with CAD (having experienced a myocardial infarction, percutaneous coronary intervention, angina pectoris, or coronary artery bypass graft surgery), symptomatic HF (New York Heart Association class I-IV), and HT were included in the study. HT was defined as systolic blood pressure ≥140 mm Hg and/or diastolic blood pressure ≥90 mm Hg on two different measurements spaced 1 to 2 min apart and after 3 to 5 min in a sitting position. The values of the second measure were used. HF patients were included if they had a diagnosis of systolic or diastolic HF and the presence of HF symptoms.

Patients were recruited at Badalona Serveis Assistencials (Badalona, Spain) and Elisabeth-TweeSteden Hospital (Tilburg, the Netherlands). The study was approved by the medical ethics committees of the participating hospitals and was conducted in accordance with the Helsinki Declaration.

The inclusion criteria were as follows: (1) a primary diagnosis of CAD, HF, or HT; (2) aged 18 to 75 years; (3) having ≥2 of the following risk factors: positive family history, increased cholesterol, smoking, diabetes, sedentary lifestyle, and/or psychosocial risk factors; (4) sufficient knowledge of the country’s native language; (5) access to the internet at home; and (6) having a smartphone compatible with the apps used in the study.

The exclusion criteria were as follows: (1) life expectancy <1 year, (2) life-threatening comorbidities (eg, malignancy), (3) history of psychiatric illness other than anxiety and/or depression, (4) significant cognitive impairments (eg, dementia), and (5) on the waiting list for heart transplantation.

### Procedure

Patients meeting the inclusion criteria were approached for participation by a cardiologist or cardiac nurse. If interested, patients received information about the study in writing and orally. After 10 days, patients were contacted to inquire about their participation. If the patient indicated that they wanted to participate, a face-to-face appointment was scheduled at the hospital. Patients were asked to sign an informed consent document and were provided with the first set of questionnaires (baseline). After filling in the questionnaires, patients were randomized. Patients in the intervention group received information about the intervention and the use of associated devices (see the *Intervention* section). The following day, patients in the intervention group were contacted by telephone to check that the devices were installed correctly and that the system was functional.

Patients received the intervention for 3 months. Follow-up questionnaires were sent at 3 and 6 months. Patients returned the devices after completion of the intervention (ie, after 3 months).

### Randomization and Blinding

Patients were randomized (1:1) after completing the baseline questionnaires. Randomization sequences were computer generated and individually sealed before recruitment started. After completing the questionnaires, one sealed envelope was drawn by the research assistant containing the group allocation. Owing to the nature of the behavioral monitoring aspects of the study, blinding health care providers or participants to the treatment condition was not possible, whereas the initial analyses of the study outcomes were analyzed without knowledge of the treatment allocation.

### Intervention

#### Do Cardiac Health Advanced New Generated Ecosystem Intervention Versus Care as Usual

##### Behavior Change Technique

Patients randomized to the intervention group received a 3-month behavior change program, *DSD*, which was provided via text messages on patients’ mobile phones. The DSD program that was used aims to change unhealthy habits through the increase of behavioral flexibility [12]. This is achieved by disrupting patients’ daily behavioral routine for a short period (few seconds) with behavioral prompts, which are referred to as *Do’s* (eg, “EXPLORE MORE DAY. Today instead of going the same old way, take a different route. Look around, spot 10 things you wouldn't see on your usual journey”) and are provided through patients’ mobile phones. These messages challenge patients to do something different and get out of their comfort zone. They have been developed by a multidisciplinary team, including cardiologists and psychologists, to make sure that the Do’s apply to the patient population and are thus related to their daily behaviors/needs. Patients received a total of 32 Do’s during the 3-month intervention period (2-3 Do’s every week). DSD has been evaluated in other patient samples and has shown promising results with respect to behavior change [13]. For this trial, the program was adapted to the cardiac population with slight differences in the program depending on patients’ primary diagnosis (eg, CAD, HF, HT), as the preferred health behaviors may vary depending on the diagnosis. For example, because of disease-specific symptoms, advice regarding fluid intake was taken into account within the program only for patients with HF. More details regarding the DSD program are provided in the previously published design paper of this project [11].

##### Technological Tools

In addition to the DSD program, to obtain objective measures on patients’ physical functioning, all patients received a blood pressure monitor, the Moves app (ProtoGeo, Helsinki; to register the GPS location), and the CarePortal (Docobo Ltd, Leatherhead; eg, a home monitoring device measuring daily symptoms and electrocardiogram). Owing to the disease-specific reasons, patients diagnosed with HF also received a weight scale, as daily weight monitoring is of importance in this subgroup. Data obtained from these devices will not be included in this analysis. This manuscript will focus on the primary outcome measures related to lifestyle parameters and patient-reported outcomes, which were derived from validated questionnaires (see the study by Habibović et al [11] for a description of primary and secondary outcomes).

##### Control Group

Patients randomized to the care as usual (CAU) group received the treatment as usual and were only provided with the validated questionnaires at baseline and at 3 and 6 months. These patients did not receive devices for ambulatory monitoring measures.

### Measures

#### Questionnaires

##### Primary outcomes

###### Behavioral Flexibility

Behavioral flexibility was measured using the *DSD* questionnaire from scale items designed for this study [11]. This scale contains 30 different descriptions of behavior coupled in 15 pairs of opposites (Multimedia Appendix 1). Patients were asked at each measurement point to select the behaviors that best describe them (eg, *gentle* or *firm*). On the basis of a formula, the behavioral flexibility for each participant at each time point was calculated as outlined below.



Every addition of behavior raises the score as well as when both of a pair of opposite behaviors are added. For example, definite, systematic, trusting, predictable, and unpredictable are selected. All these selected behaviors raise the flexibility score. However, because predictable and unpredictable are each other’s opposites, these are added to the formula again and increase the flexibility score even more. The model interprets this seemingly contradictory behavior as evidence of flexibility: based on what a given situation demands, the person can use different reactions and thus be more flexible. The total score can range from 0 to 100. The internal consistency in this sample was considered acceptable (Cronbach alpha=.67 to .76).

###### Lifestyle

The Health-Promoting Lifestyle Profile questionnaire was administered to assess health-promoting lifestyle habits [14]. This survey evaluates whether the subjective perception of patients regarding their lifestyle is changed and consists of 52 items (eg, “Eat 6-11 servings of bread, cereal, rice, and pasta each day”) in total. Each item can be answered on a 4-point Likert scale, ranging from 1 (never) to 4 (routinely). The total score can therefore range from 52 to 208, with a higher score indicating a better lifestyle. Furthermore, the questionnaire includes 6 different subscales that each cover a health promotion lifestyle domain (ie, Physical Activity, Spiritual Growth, Health Responsibility, Interpersonal Relationships, Nutrition, and Stress Management). The internal consistency was considered as excellent in this sample (Cronbach alpha=.88 to .90).

###### Quality of Life

To administer changes in the quality of life, participants completed the World Health Organization Quality of Life—BREF (WHOQOL-BREF) [15]. The WHOQOL-BREF is considered a reliable, generic multidimensional quality of life measure and consists of 26 items in total. Two items refer to the facet’s overall quality of life and general health, whereas the abiding 24 items reflect 4 different domains (ie, physical health, psychological health, social relationships, and environment). The internal consistency in this sample was excellent (Cronbach alpha=.89 to .90).

###### Perceived Usefulness and Acceptance

The Unified Theory of Acceptance 2 (UTAUT2) scale [16] was administered to assess the perceived usefulness and acceptance of the tools that were used in the intervention. Mean scores on 8 subscales are provided, namely, (1) Performance Expectancy, (2) Effort Expectancy, (3) Social Influence, (4) Facilitating Conditions, (5) Hedonic Motivation, (6) Habit, and (7) Behavioral Intention. The initial subscale, *Price Value*, of the UTAUT2 was not included, as the cost per individual for the intervention could not be estimated. The total score per subscale can range from 4 to 20, with a higher score indicating higher usefulness and acceptance [16]. The internal consistency in this sample was excellent (Cronbach alpha=.89).

###### Client Satisfaction Questionnaire

To assess the satisfaction of the patients about the ecosystem, the 8-item Client Satisfaction Questionnaire [17] was used. This self-administered questionnaire is a general scale that consists of 8 Likert scale items (eg, “To what extent has our program met your needs?”) ranging from 0 to 4, with response descriptors that vary. The overall score can range from 8 to 32, with a higher score indicating a higher satisfaction. The internal consistency was rated as excellent (Cronbach alpha=.92) [17].

#### Other Questionnaires Included in the Model

##### Type D Scale (Distress Scale-14)

Type D personality was assessed using the Type D scale (Distress Scale-14) [18]. This 14-item questionnaire consists of 2 subscales with seven 5-point Likert scale items each, ranging from 0 (false) to 4 (true). Total scores on both subscales range from 0 to 28. The 2 subscales represent the characteristics of negative affectivity (NA; eg, the tendency to experience negative emotions across time and situations) and social inhibition (SI; eg, the tendency not to express feelings). When scoring ≥10 on both subscales, patients were classified as Type D. With a reported Cronbach alpha value of .86 and .88, respectively, the internal consistency of SI and NA are considered as satisfactory [18].

##### The 7-Item Generalized Anxiety Disorder Scale

To gauge self-administered symptoms of anxiety, the 7-item Generalized Anxiety Disorder scale was administered [19]. The questionnaire is comprised of 7 items (eg, “Feeling afraid as if something terrible might happen”) that can be answered on a 4-point Likert scale, ranging from 0 (not at all) to 3 (almost every day). To get an indication of anxiety symptom severity, the total score (range from 0 to 21) can be used. A higher score implies higher levels of anxiety. The internal consistency was considered excellent (Cronbach alpha=.92) [19].

##### Nine-Item Patient Health Questionnaire

Depressive symptoms were administered at each time point by using the 9-item Patient Health Questionnaire (PHQ-9) [20]. This self-report questionnaire consists of 9 items in total (eg, *feeling down*, *depressed*, or *hopeless*), each evaluated on a 4-point Likert scale (ie, not at all, several days, and nearly every day). The total score ranges from 0 to 27, with a higher score as an indication of worse depression symptom severity. The internal consistency was considered excellent (Cronbach alpha=.90) [21].

#### Demographic and Clinical Data

Demographic characteristics (eg, age, sex, marital status, working status, level of education, and smoking behavior) were obtained by patients’ self-report. Clinical data (eg, comorbidities; prescribed cardiac medication; prescribed psychotropic medication; left ventricular ejection fraction; history of coronary artery bypass grafting; history of percutaneous coronary intervention; and resting heart rate and systolic and diastolic blood pressure measured at the most recent outpatient visit) were obtained from the medical record.

#### Statistical Analyses

Categorical variables were compared using chi-square tests, and continuous variables were compared using a two-tailed *t* test for independent samples. To evaluate the treatment effectiveness, based on intention-to-treat, a univariate and multivariate Linear Mixed Model analysis was performed. Multivariable analyses were adjusted for age, sex, education, site of inclusion (Badalona Serveis Assistencials [BSA] or Elisabeth-TweeSteden Hospital [ETZ]), primary diagnosis, Charlson comorbidity index scores, Type D personality, baseline anxiety scores, and baseline depression scores. *F* values with two-sided *P* values were reported for main and interaction effects (group×time). For the estimated fixed effects, beta coefficients with two-sided *P* values were reported. Data were analyzed using the SPSS software package (version 24).

## Results

### Sample

**Figure 1:**
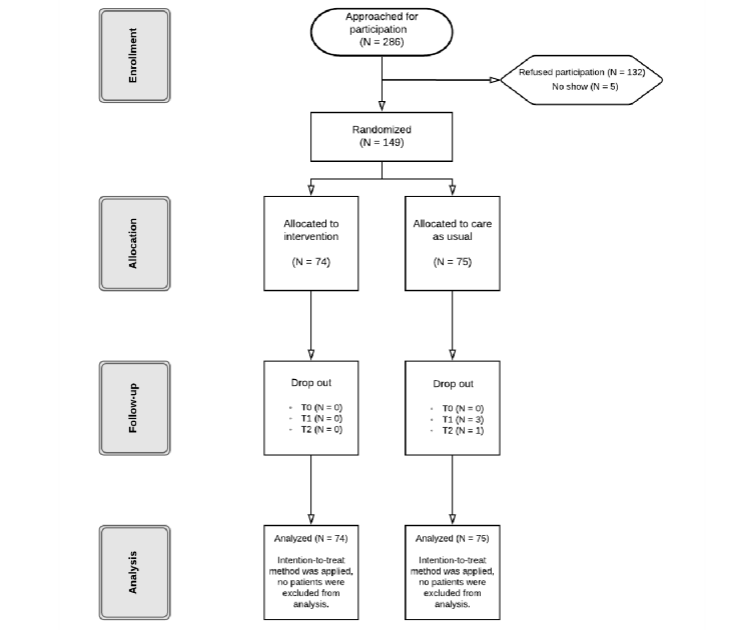
Flow diagram of patient recruitment.

The data collection took place between January 2017 and September 2017. In total, 286 eligible patients were approached for participation, of which 132/286 (46.1%) patients refused to participate. An additional 5/286 (1.7%) participants did not show up or declined participation. The reasons for refusal included that it would be too time consuming, they did not want to be confronted about their heart disease every day, and they were reluctant to use technology. A total of 149 (response rate: 149/286; 52.0%) patients were enrolled. Enrollment per study site was as follows: BSA randomized a total of 74 (intervention: n=37 and CAU: n=37) patients; ETZ randomized 75 patients (intervention: n = 37 and CAU: n=38). Of the total sample, 4 participants within the CAU condition dropped out, as they did not receive the intervention and were therefore not willing to continue. Of the patients randomized to the intervention condition, 82% (61/74) reported having completed the entire 3-month program. Overall, 97.3% (145/149) of the participants completed the follow-up assessments. Figure 1 presents the flowchart of patient recruitment.

### Baseline Characteristics

The mean age of the total sample was 63.6 (SD 8.3) years, and 66% (99/149) were men. There were significant differences observed in mean completed education in years between the intervention group (mean 14.3, SD 6.2) and the CAU group (mean 11.8, SD 7.9; *P*=.03). This means that patients in the intervention group completed more years of education than those in the CAU group. Furthermore, a significant difference between the 2 groups was found on the mean PHQ-9 baseline scores, with a higher mean score on depressive symptoms in the CAU group (mean 3.61, SD 3.6 vs mean 5.56, SD 4.17; *P*=.003). No other differences were found between the intervention and CAU groups. Table 1 presents an overview of the baseline characteristics of this sample.

**Table 1:** Baseline patient characteristics of the total sample.

Variable		Total	Do Cardiac Health Advanced New Generation Ecosystem intervention (N=74)	Care as usual (N=75)	*P* value
**Site of allocation, n (%)**					
	Badalona Serveis Assistencials	74 (49.7)	37 (50)	37 (50)	N/A^a^
	Elisabeth-TweeSteden Hospital	75 (50.3)	37 (49.3)	38 (50.7)	N/A
	Total	149 (100)	74 (49.7)	75 (50.3)	N/A
**Demographics**					
	Age (years), mean (SD)	63.57 (8.30)	63.26 (8,35)	63.88 (8.30)	.65
	Gender (male), n (%)	99 (66.4)	52 (70.3)	47 (62.7)	.42
	Education (years), mean (SD)	13.03 (7.22)	14.30 (6.24)	11.79 (7.91)	.03
	Marital status (partner), n (%)	118 (79.2)	61 (82.4)	57 (76.0)	.44
	Working status (working), n (%)	55 (36.9)	28 (37.8)	27 (36.0)	.95
	Smoking (yes), n (%)	27 (18.1)	10 (13.5)	17 (22.7)	.29
**Clinical**					
	Diagnosis heart failure, n (%)	36 (24.2)	21 (28.4)	15 (20.0)	.96
	Diagnosis hypertension, n (%)	73 (49.0)	38 (51.4)	35 (46.7)	>.99
	Diagnosis coronary artery disease, n (%)	40 (26.8)	15 (20.3)	33 (33.3)	.07
	Charlson comorbidity index, mean (SD)	1.14 (0.95)	1.01 (0.88)	1.27 (1.00)	.11
	Systolic blood pressure (baseline), mean (SD)	138.02 (19.71)	135.00 (20.89)	141.00 (18.13)	.06
	Diastolic blood pressure (baseline), mean (SD)	79.27 (10.01)	78.76 (10.33)	79.77 (9.87)	.54
	Heart rate (rest), mean (SD)	69.41 (11.97)	69.95 (14.43)	68.88 (11.57)	.59
**Psychological**					
	Patient Health Questionnaire-9, mean (SD)	4.59 (4.00)	3.61 (3.60)	5.56 (4.17)	.003
	Generalized Anxiety Disorder-7, mean (SD)	4.03 (4.37)	3.35 (4.07)	4.69 (4.59)	.06
	Type D personality (yes), n (%)	38 (25.5)	20 (27.0)	18 (24.0)	.67

^a^N/A: not applicable.

### Intervention Effects

#### Behavioral Flexibility

**Figure 2:**
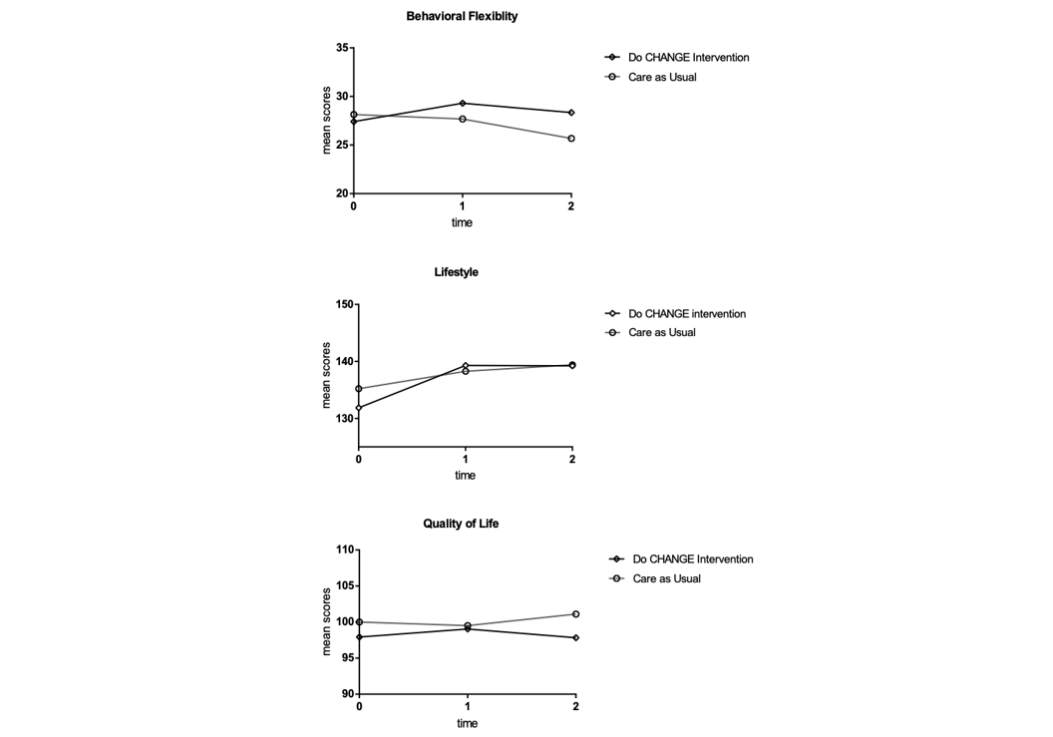
Mean scores of intervention and care as usual group on primary outcome measures.

The univariate analysis on behavioral flexibility scores (including group, time, and group×time) revealed no significant main effects for group (*F*_1,148.93_=3.42; *P*=.07) or time (*F*_2,146.82_=1.69; *P*=.18) and group×time interaction (*F*_2,146.82_=1.09; *P*=.34). After adjusting for covariates (as previously described), main effects for time (*F*_1,146.81_=1.74; *P*=.18), group (*F*_1,149.00_=0.48; *P*=.07), and group×time (*F*_2,146.81_=1.13; *P*=.33) remained nonsignificant (Figure 2). These findings indicate that behavioral flexibility scores did not significantly change over time, and that there were no differences between the 2 groups. With regard to covariates included in the model, the estimated fixed effects of HT (β=−6.07; *P*=.01) and CAD (β=−5.57; *P*=.02) were significantly associated with lower levels of behavioral flexibility scores (across all time points). In addition, the site of recruitment was associated with behavioral flexibility scores, with only patients from Spain showing an increase in behavioral flexibility over time (β=6.31; *P*<.01) when compared with those in the Netherlands (see Table 2).

**Figure 3:**
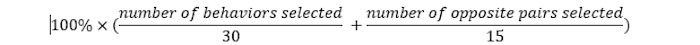
Standalone Equation 1.

**Table 2:** Estimates of fixed effects from multivariable linear mixed models on the main outcome measures at baseline and at 3- and 6-month follow-up.

Multivariable linear mixed model		Behavioral flexibility			Lifestyle				Quality of life			
		Estimate	SE	*P* value		Estimate	SE	*P* value		Estimate	SE	*P* value
**Model 1: unadjusted model**												
	Intervention group	4.70	2.07	.03		2.18	3.52	.54		0.51	1.89	.79
**Model 2: covariates adjusted model**												
	Intervention group	2.91	1.99	.15		0.46	3.27	.89		−2.89	1.69	.09
	Hypertension^a^	−6.07	2.27	.01		−3.89	3.72	.30		−3.40	2.07	.10
	Coronary artery disease^a^	−5.57	2.29	.02		3.62	3.74	.33		−2.02	2.08	.33
	Study site (Spain)	6.31	1.84	<.01		12.86	3.01	<.01		4.62	1.67	<.01
	Sex (male)	2.36	1.79	.19		−1.66	2.93	.57		2.66	1.63	.11
	Type D	−0.28	2.14	.90		8.33	13.47	.54		−10.85	7.48	.15
	Age (years)	0.08	0.09	.42		−0.04	0.16	.80		0.07	0.09	.45
	Higher Charlson comorbidity index	−1.73	1.00	.08		−1.09	1.63	.51		−1.76	0.90	.05
	Higher education	−0.01	0.12	.95		0.08	0.19	.66		0.25	0.11	.02
	Anxiety	-0.12	0.44	.79		−1.38	0.72	.06		-0.90	0.40	.03
	Depression	−1.77	1.13	.12		−4.17	1.86	.03		−3.63	1.03	<.01

^a^Compared with the main diagnosis of heart failure.

#### Lifestyle

A univariate analysis showed no significant effect for group (*F*_1,434.91_=0.91; *P*=.34) or group×time (*F*_2,282.73_=0.39; *P*=.68). These findings present that, without the addition of possible confounding variables, the intervention and CAU group did not differ. In addition, no interaction effect between allocation to group and time was found. However, a significant improvement for both groups on overall reported lifestyle behavior was found (*F*_2,282.73_=4.28; *P*=.02). When adjusting for covariates in the multivariable analysis, this improvement remained significant (*F*_2,146.63_=9.99; *P*<.001). As shown in Figure 2, both groups reported improvements in lifestyle behavior over time. The effects of interaction (*F*_2,147.02_=1.36; *P*=.26) and allocation to group (*F*_1,149.90_=0.09; *P*=.77) remained nonsignificant in the adjusted models. This indicated that no effect of the intervention on healthy lifestyle behavior was found. The estimated fixed effect of depression (β=−4.17; *P*=.03) was negatively associated with lifestyle promoting behavior, indicating that patients who score higher on the depression scale report lower healthy lifestyle behaviors. Patients from Spain showed an increase in lifestyle behavior (β=12.86; *P*<.01), in comparison with those in the Netherlands (see Table 2).

#### Quality of Life

The results of the univariate analysis of the quality of life total scores showed an interaction effect between time and group (*F*_2,146.40_=4.22; *P*=.02). This finding indicates that the mean scores on quality of life of the intervention and CAU groups have different slopes over time: the intervention group showed a small improvement in the quality of life after 3 months, whereas the CAU group reported a small decline in the quality of life (mean improvement 1.11, SD .11 vs mean −1.47, SD .11). Both groups stabilized to baseline level after 6 months. The interaction effect remained significant after adding the covariates in the multilevel analysis (*F*_2,146.52_=4.29; *P*=.02; Figure 2), suggesting a significant, positive effect of the intervention on self-reported quality of life in the first 3 months. The estimated fixed effects of higher levels of education (β=.25; *P*=.02) and being recruited in Spain (β=4.62; *P*=.008) compared with those in the Netherlands were significantly associated with higher scores on quality of life. Lower scores were predicted by higher CCI scores (β=−1.76; *P*=.05) anxiety (β=-0.90; *P*=.03), and depression (β=−3.63; *P*<.01; see Table 2). Examining subscales of the WHOQOL revealed no specific subscale differences regarding response patterns to the intervention.

### Acceptability and Satisfaction

Overall, patients in the intervention group indicated being satisfied with the intervention (mean 26.22, SD 4.82). The intervention was perceived to be useful (mean 13.88, SD 3.96) and easy to use (mean 17.07, SD 2.57). Patients did not feel social pressure to use the devices from the intervention (mean 9.85, SD 3.63) and reported to be quite satisfied with the possibilities to receive support (mean 15.44, SD 2.43) and had a neutral opinion regarding the pleasure in using the devices offered in the intervention (mean 10.63, SD 2.44). Furthermore, the intervention was integrated relatively well in patients’ lives (mean 11.71, SD 3.05). However, patients indicated that they were neutral regarding the intention to use the ecosystem in the future (mean 8.40, SD 3.34).

## Discussion

### Principal Findings

This study aimed to provide proof of concept for the Do CHANGE behavioral intervention targeting behavioral flexibility, lifestyle change, and quality of life in cardiac patients. No significant differences between the groups were observed on behavioral flexibility and lifestyle. However, a small increase in quality of life at 3 months was observed in the intervention group, but at 6 months, no significant difference between the groups was observed. With respect to the usefulness and feasibility of the intervention, the findings of this study revealed that the ecosystem is experienced as useful, easy to use, and integrated well into the daily life of the patients. It made the participants more aware of the fact that they must undertake activities themselves to feel better. Patients also reported feeling more *safe* because health care professionals were watching along. Nonadherence is a common issue in Web-based interventions for promoting health-related behavior, and the average study results in only 50% of participants adhering to the intended intervention [22]. However, 82.4% of the patients participating in this Do CHANGE intervention condition completed the intervention, which may further indicate that the intervention was not perceived as demanding.

The findings of the study are not completely in line with previous studies in other patient populations [13]. An explanation for this discrepancy could be the fact that this was the first study implementing the concept of behavioral flexibility and thus the core Do’s of the DSD program in the cardiac population. In addition, the Do’s might not have been tailored enough to the patients’ needs that the timing of the Do’s might not have been optimal. For example, one would want a patient to receive a distractive Do at the time when the *unwanted* behavior occurs. In this trial, patients from 2 different cultures (Spain and the Netherlands) and diagnosed with different cardiac disorders (ie, HT, HF, or CAD) were enrolled. This reflects the heterogeneity of the sample, which may have affected the results. Another important area for future research is the exploration of the mediating factors that drive the interplay between behavioral flexibility, lifestyle factors, and quality of life in the setting to electronic health (eHealth) interventions.

Enrollment in the study may have increased the general awareness of lifestyle change in both groups. This awareness could unknowingly lead to the adaptation of a lifestyle, independent of the allocation to a group. Previous research in cardiac patients affirms that there is a relationship between general knowledge about cardiac risk factors and self-reported lifestyle changes in the short term [23]. Furthermore, although lifestyle change is crucial in the treatment of cardiovascular disease, there is a lack of emphasis on lifestyle change and self-care of the patient in the current health care systems [24]. Addressing self-care and lifestyle change in clinical practice is therefore warranted. The Do CHANGE trial provides (longitudinal and momentary) knowledge that can be used in the further development of personalized interventions that will help patients reach recommended lifestyle goals.

Behavioral flexibility is an important construct on which behavioral change can possibly be initiated. However, the results of this study may indicate the need for a better measurement tool, as the questionnaire that is used might not be sensitive enough to reveal significant alteration in patients’ behavioral flexibility over time.

The findings on quality of life, on the other hand, are not entirely in line with previous studies in cardiac samples, which have shown that there is a decline in quality of life and generally a slight increase in anxiety and depression scores within the 3 months postcardiac event [25]. This could be explained by patients having to adapt to new behaviors after visiting the hospital and being reminded of the fact that they have a chronic illness. In this study, the intervention group received the behavioral program, which could have contributed to first an increase in their quality of life, with a slight decrease after 3 months, sustaining their baseline quality of life. After the behavioral intervention ends, the quality of life in the intervention group also goes down as the additional *support* is no longer provided.

The findings of this study must be interpreted in light of a few limitations. At baseline, the intervention group and CAU group showed some differences in mean years of completed education and mean scores on depressive symptoms. The CAU group scored significantly higher on both variables. Another limitation of this study was that the sample was rather small, in relatively good health, and clinically heterogeneous, which may have limited the possibility to find substantial effects. Although the intervention was positively evaluated by participants, half of the approached patients did refuse participation. Therefore, it can be concluded that eHealth interventions similar to those described in this study are appealing for certain subgroups of patients. Future research should focus on eHealth interventions within the cardiac population based on a larger sample with significant power that is assessed over a prolonged follow-up duration (eg, beyond 6 months) to draw firm conclusions on sustainable behavior change. The results of this study showed that depression was associated with negative behavioral and psychological outcomes, which is in line with previous findings [26]. Depressive symptoms are common in patients with cardiovascular disease [27-29] and are related to various behavioral risk factors (eg, sedentary lifestyle, unhealthy diet, alcohol overconsumption, and smoking) [30,31]; this may explain the relation between depression and lower lifestyle behavior scores. Hence, future research is needed to examine which psychological and clinical factors contributing to health behavior change and potentially address these factors during the intervention.

For clinical practice, it is important to acknowledge that technology and eHealth solutions might be the feasible way forward in meeting patient needs and initiated health behavior change. However, the findings of this study underline the importance of a personalized approach that includes the assessment of a patient’s demographic, clinical, and psychological profile.

### Conclusions

In conclusion, the Do CHANGE 1 intervention was perceived as useful and easy to use. However, no main effects were found on behavioral flexibility, lifestyle behavior, and quality of life. More research is warranted to examine which components of behavioral interventions, and in which patients, are effective in producing long-term behavior change.

## Appendix

Multimedia Appendix 1 Consolidated Standards of Reporting Trials of Electronic and Mobile HEalth Applications and onLine TeleHealth checklist.

Multimedia Appendix 2 CONSORT-EHEALTH checklist (V 1.6.1).

## Abbreviations

CAD: coronary artery disease
CAU: care as usual
Do CHANGE: Do Cardiac Health Advanced New Generation Ecosystem
DSD: Do Something Different
eHealth: electronic health
HF: heart failure
HT: hypertension
NA: negative affectivity
PHQ-9: Patient Health Questionnaire 9
SI: social inhibition
UTAUT2: Unified Theory of Acceptance 2
WHOQOL-BREF: World Health Organization Quality of Life—BREF
