# Supplementary figures and images for: A Personalized eHealth Intervention for Lifestyle Changes in Patients With Cardiovascular Disease: Randomized Controlled Trial
Source: J Med Internet Res. 2020 May 22;22(5):e14570. doi: 10.2196/14570 (PMC7381027; doi:10.2196/14570)

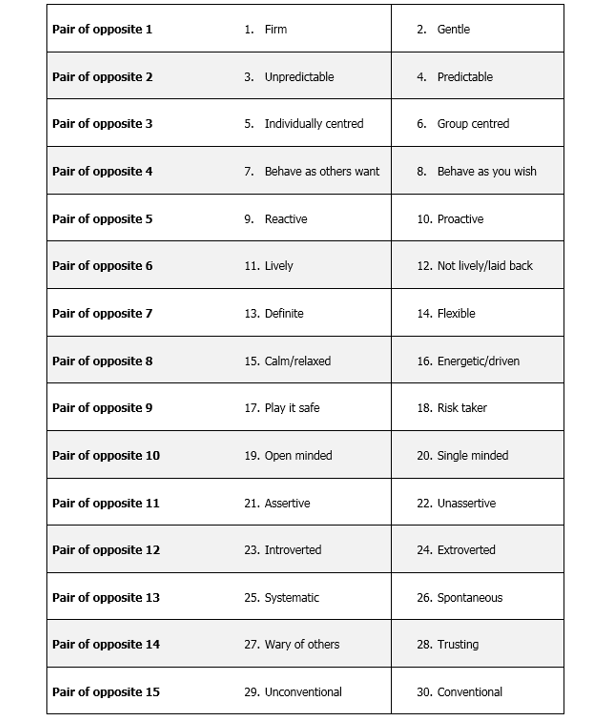

Supplement: Multimedia Appendix 1 [file jmir_v22i5e14570_app1.PNG]
